# Long Life Family Study Participants Carry Rare Variants That Correlate With Better Cognitive Function

**DOI:** 10.1093/geroni/igaf122.3805

**Published:** 2025-12-31

**Authors:** Hannah Lords, Mengze Li, Zeyuan Song, Anastasia Leshchyk, Harold Bae, Stacy Andersen, Anastasia Gurinovich, Paola Sebastiani

**Affiliations:** Boston University, Boston, Massachusetts, United States; Boston University, Boston, Massachusetts, United States; Tufts Medical Center, Boston, Massachusetts, United States; Tufts Medical Center, Boston, Massachusetts, United States; Oregon State University, Corvallis, Oregon, United States; Boston University, Boston, Massachusetts, United States; Tufts Medical Center, Boston, Massachusetts, United States; Tufts Medical Center, Boston, Massachusetts, United States

## Abstract

Cognitive impairment is a growing healthcare and quality-of-life challenge as more individuals reach older ages. Without effective interventions, the prevalence of decline will continue to rise. To identify genetic factors underlying cognitive function, we performed GWAS on nine neuropsychological test scores in the Long Life Family Study (LLFS). We then compared results with UK Biobank GWAS of general cognitive ability and reaction time, conducted quantitative trait locus (QTL) analyses of whole blood transcriptomic and serum metabolomic data for genome-wide significant variants (p < 5e-8), and performed gene set enrichment analyses of nominally significant transcripts. We identified 12 genome-wide significant variants across 7 tests in LLFS. Comparison with UK Biobank data revealed test-specific replication patterns, suggesting that loci for general cognitive function and reaction time reflect distinct cognitive domains. QTL analyses identified one SNP–transcript association (p < 0.05/16,300) but no significant SNP–lipid or SNP–polar metabolite associations. Three rare protective variants—rs190287985, rs75730801, and rs552842447—were linked to semantic fluency (animal), phonemic fluency (FAS), and digit span forward, respectively. Two rare deleterious variants, rs556333682 and rs188304645, associated with performance on the Hopkins Verbal Learning Test (HVLT) total recall, lie in ischemia-related genes SH3TC1 and RPH3A. Another variant, rs180691759, associated with HVLT delayed recall, was proximal to RSPO3 and linked to decreased ECHDC1 expression, implicating ischemic and unexplained ethylmalonic acid pathways. Gene set enrichment identified three pathways (FDR < 0.05), including KEGG JAK-STAT signaling and oxidative phosphorylation. These findings highlight domain-specific mechanisms of cognitive resilience and decline.

